# Harvey Cushing: early use of tendon transfers for repair of foot deformity: A historical case report

**DOI:** 10.3109/17453674.2011.596065

**Published:** 2011-09-02

**Authors:** Courtney Pendleton, Alfredo Quinones-Hinojosa, Richard J Redett, Amir H Dorafshar

**Affiliations:** ^1^Department of Neurosurgery; ^2^Department of Plastic and Reconstructive Surgery, Johns Hopkins Medical Institute, Baltimore, MD, USA; Correspondence: adorafs1@jhmi.edu

## Abstract

We describe 4 cases of tendon transfers for correction of foot deformities, which were performed by Harvey Cushing in 1898.

Lower extremity tendon transfers were first described in the literature in the late nineteenth century, with European surgeons contributing to the development of the technique. Tendon transfer was initially described in the treatment of post-poliomyelitis paralysis (Jeng and Myerson 2004), but near the start of the twentieth century tendon transfers were developed for the correction of congenital talipes equina (Codavilla 1976, Jeng and Myerson 2004).

Around this time, Harvey Cushing was developing his surgical practice at the Johns Hopkins Hospital. Before he concentrated on neurological surgery, he performed a number of procedures for the correction of congenital and acquired equinus deformity. Here, we describe his operative technique in 4 of these cases, performed in 1898.

Following IRB approval, and through the courtesy of the Alan Mason Chesney Archives, we reviewed the surgical records for the Johns Hopkins Hospital, 1896 to 1912. Approximately 26,000 surgical files were reviewed, and the records of those patients who underwent operative intervention by Cushing were selected for further analysis. Four representative cases of tendon transfer for the correction of foot deformity were chosen from this set of records and are reported here.

## Case 1

On March 9, 1898, a 6-year-old boy presented with congenital talipes equina varus. Cushing brought him to the operating room on March 15 for an operation entitled “tendon transplantation for relief of paralytic club foot.”

“Tenotomy of tendon Achilles does not allow foot to be well corrected and to remain so. Evidently the muscles to the inner side of the foot are under greater tension than those to the outer side. This seems to be chiefly due to the proprius pollicis extensor which holds the toe extended in marked hammer toe. Transplantation of tendon deemed advisable.

**Figure 1.**
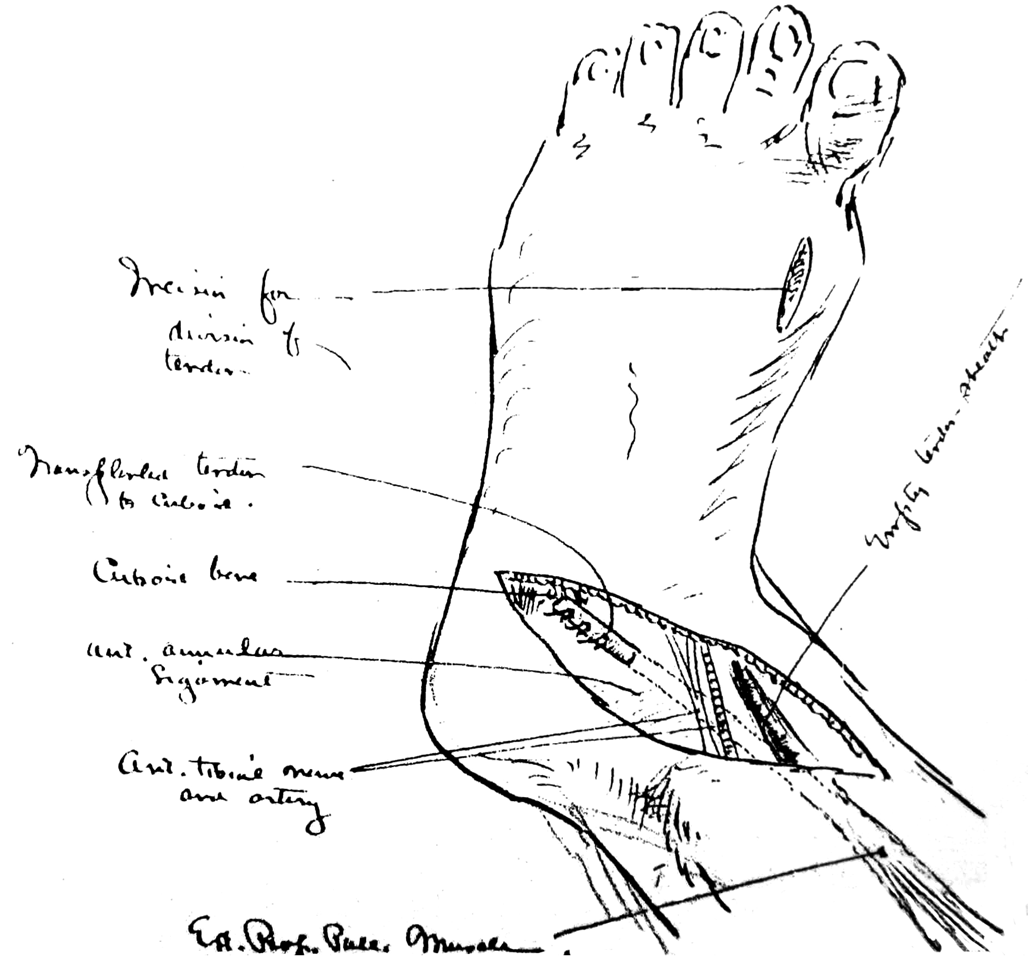
Part of Cushing's operative illustrations documenting the tendon transfer procedures. Case 1. The extensor proprius pollicis was released from its attachment through an incision on the dorsum of the foot, and sutured to the periosteum of the cuboid bone.

Oblique incision made from cuboid bone across ankle joint to position of long extensor tendon (Figure 1).

The tendon sheath of long extensor of great toe was opened and tendon drawn up. A small incision was made over the tendon in the foot, proximal to the junction with the small extensor. Tendon was divided there and drawn up into the ankle wound.

A grooved dissector was then passed from the empty tendon sheath under the ant. annular ligament and [therefore] under the artery and nerve which were uninjured. A large probe with large eye was then passed through, the tendon threaded in this eye, and passed under the ligament.

The end of the tendon was then sutured by 3 mattress sutures to the cuboid periosteum and extensor of the peroneus brevis muscle. The attachments held the foot in natural position without support. Wounds closed with silver wire. Foot put up in plaster.”

The patient was discharged on postoperative day 6. A letter from the patient's father, dated 18 August, 1898, said that the child “is doing well and in my opinion is acquiring better use of his feet. He plays baseball and uses it with much more naturalness than formerly.”

## Case 2

On March 19, 1898, a 6-year-old presented with “talipes equines paralytic.” Cushing brought the child to the operating room on April 16:

“Tenotomy (subcutaneous) of the tendo achilles. This allowed flexion of the foot on the leg but the flexion scarcely reached a r. angle probably because of deformity of bones.

An oblique incision over the instep in site of annular ligament. This exposed the tendon of the tibialis anticus which was rather small. Also exposed the dorsal veins these left intact – also the tibialis ant. nerve. A little dissecting brought the musculo-tendinous junction of the extensor proprius pollicis into view. This muscle thick and well developed.

**Figure 2.**
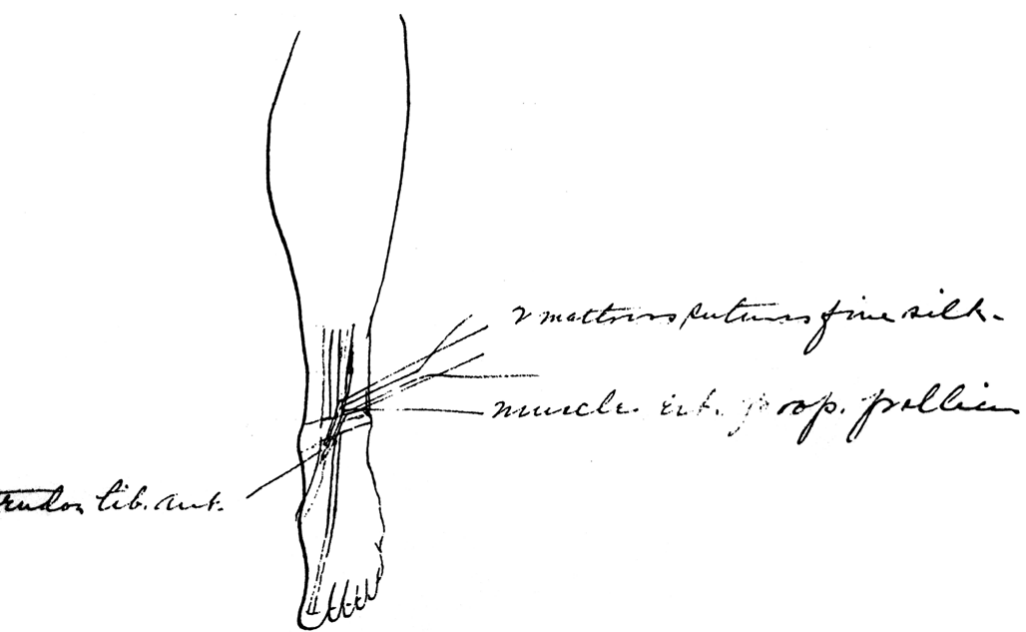
Part of Cushing's operative illustrations documenting the tendon transfer procedures. Case 2. The extensor proprius pollicis was removed from its distal attachment, and sutured to the tendon tibialis anterior.

With the foot flexed on the leg the muscle of the ext. prop. pollicis was drawn down and the tendon of the tib. ant. drawn up – the two laid side by side and without cutting, sutured in this shortened position with 2 mattress sutures of fine silk on interstitial needles. Skin closed with silver wire. Dressing silver foil-plastic.” (Figure 2).

The patient was discharged on postoperative day 63. Cushing noted:

“The foot has considerable […] flexion but is dragged along floor in walking. Does not lift it off the floor in walking. The foot cannot be flexed quite to a right angle with leg. The foot is readily inverted, not readily everted.”

## Case 3

On May 2, 1898, a 29-year-old male presented with “talipes equine varus.” The admission note documented:

“both legs are very much atrophied and thigh slightly so [...] the right foot shows a scar on outer side just below and anterior to malleolus [...] the scar is the result of a blow from an axe when patient was nine years old and to which he attributes all his trouble although for a year after the accident he was able to move easily and naturally.”

He was brought to the operating room on May 11 for a tendon anastomosis.

“I. tenotomy of tendon Achilles. II incision across foot from cuboid to site of extensor proprius tendon above ant. annular ligament. This tendon exposed. III. Incision over site of this tendon just proximal to site of attachment of ext. brevis pollicis. Tendon divided at this point and drawn out of wound above annular ligament.

Tendon then passed under ant. annular ligament emerging just at prominence of cuboid where it was sutured to the periosteum of the cuboid and to the exposed tendon of the peroneus brevis muscle.

Foot stayed in excellent position after the transplantation. The tendon of transplanted muscle ran smoothly in its new bed.

Wounds closed with silver wire.

Plaster holding foot in over corrected position.”

Cushing documented the patient's postoperative course. On May 23:

“The plaster removed and foot found in good position. The wounds healed per primam. Sutures removed.

Foot can be flexed without the inward motion, and can be held by patient in flexed position.

Put back in plaster, and discharged to be treated by his local physician.”

No further follow-up information was available.

## Case 4

On October 31, 1898, a 14-year-old boy presented with “acquired club foot. Talipes Equina.”

**Figure 3.**
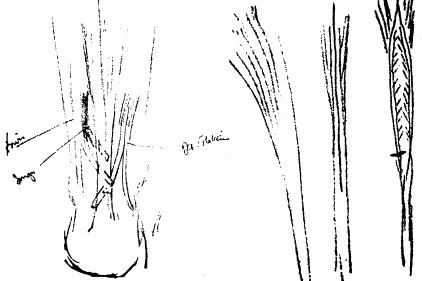
Part of Cushing's operative illustrations documenting the tendon transfer procedures. Case 4. Left: The extensor proprius pollicis and tibialis posterior were sutured to the Achilles tendon. Right: The split tendon method.

He was brought to the operating room on November 16 for bilateral operations (Figure 3):

“Right Foot.

Subcutaneous tenotomy of tendo Achilles which allowed the foot to be held almost at a right angle. Longitudinal incision (12 cm) along middle of dorsum of foot and lower half of ant portion of leg. Lower part of belly of tibialis anterior muscle exposed and found to be pale (dry leaf appearance) characteristic of paralysis. Tendon and part of muscle of ext long hallucis exposed muscle deep red (normal).

(Ex. Long hallucis) muscle was divided about quarter of way toward origin and the corresponding tendon split longitudinally down to near ant. annular ligament when it was cut off. The splice was brought into edge of ext tendon of tibialis anterior tendon, the appearing surfaces […] united by interrupted silk sutures. The foot being held in dorsal flexion. This produced a flaccid condition of the intact section of the ext. long hallucis – which is expected.”

Left Foot

Subcutaneous division of plantar fascia – Long longitudinal incision about 20cm over tendo-achilles from insertion up gastrocnemous (lower part) exposed and found to be pale brown (paralyzed). The perronei muscles brevis and longus were exposed. Dark red (active) and split longitudinally with their tendons as far as ext. malleolus where the split portion was divided. The half tendons of each muscle were brought over and threaded through incisions made in the tendo-achilles.

The muscle and tendon of tibialis posterior were likewise longitudinally spit on the inner side and its tendon splice brought through in a third hole in the tendon Achilles.

The foot was then held in corrected position and the tendons sutured in their holes by interrupted through and through silk suture.”

The patient was discharged on January 17, 1899, following a 79-day admission. A follow-up letter dated March 14, 1899 described the patient's condition:

“Left foot. When I left the Hospital there was a scab on it, the scab came off and it discharged some matter, another scab formed and yesterday it discharged a little matter but none today, and looks as if it is going to heal up.

There is not any soreness to the touch.

I can stand on my tip toes, with the help of the right foot and can move my foot inward […] with ease. The circumference five inches below the knee cap is eleven and seven-eighth inches. I still wear a bandage.

Right foot: Wounds are allright [sic]. I can move my foot upward and outward and downward but cannot move it but a very little bit inward. The circumference five inches below the kneecap is eleven and one half inches.

Both feet are a great deal stronger now than they were when I left the Hospital and are still improving. I think I have improved in my walking and feel a great deal stronger on my feet.”

No further follow-up information was available.

## Discussion

The first description of a tendon transfer was written by Nicoladoni in 1881 (Jeng and Myerson 2004), for an adolescent patient with post-poliomyelitis paralysis. In this case, a tendon transfer from the peroneus musculature to the Achilles tendon was performed. Later, Drobnek advocated the use of split-tendon technique (Mann 1972) as a refinement of the initial total tendon transplantation, in order to preserve function (Codavilla 1976, Jeng and Myerson 2004). Codavilla, an Italian surgeon and director of the Rizzoli Institute of Bologna, described the use of lower extremity tendon transfer for the correction of congenital equina deformity in 1899 (Codavilla 1976, Jeng and Myerson 2004).

Interestingly, Cushing, in 1898, one year before Codavilla's publication, performed—in what appear to be 4 separate cases of extensor hallucis longus (previously referred to as extensor proprius pollicis, according to Cushing's operative notes)—transfer to the cuboid bone, for correction of congenital equina deformity. In case 4, Cushing performed a bilateral procedure, with the right side using the technique described above and the left side employing a split-peroneus tendon to Achilles tendon transfer technique, as described by Drobnek at around the same time. Another point of interest is the technique Cushing employed of inserting the transplanted tendon directly into the periosteum of the cuboid bone; it was quite novel at the time, although subsequently it has proven to be less efficacious than tendon-to-tendon transfers described by his contemporaries. Nevertheless, the cases described here demonstrate that Cushing was an early participant in the development of the surgical treatment of congenital equina foot deformity.
